# Reduced corneal nerve fibre length in prediabetes and type 2 diabetes: The Maastricht Study

**DOI:** 10.1111/aos.14359

**Published:** 2020-02-03

**Authors:** Eline E.B. De Clerck, Jan S.A.G. Schouten, Tos T.J.M. Berendschot, Renée S. Koolschijn, Rudy M.M.A. Nuijts, Miranda T. Schram, Nicolaas C. Schaper, Ronald M.A. Henry, Pieter C. Dagnelie, Alfredo Ruggeri, Pedro Guimarães, Coen D.A. Stehouwer, Carroll A.B. Webers

**Affiliations:** ^1^ University Eye Clinic Maastricht Maastricht University Medical Center + Maastricht the Netherlands; ^2^ Department of Internal Medicine Maastricht University Medical Center + Maastricht the Netherlands; ^3^ CARIM School for Cardiovascular Diseases Maastricht University Maastricht the Netherlands; ^4^ CAPHRI School for Public Health and Primary Care Maastricht University Maastricht the Netherlands; ^5^ Department of Epidemiology Maastricht University Maastricht the Netherlands; ^6^ Department of Information Engineering University of Padua Padua Italy

**Keywords:** corneal confocal microscopy, corneal nerves, type 2 diabetes, prediabetes

## Abstract

**Purpose:**

In individuals with diabetes, injury to the corneal nerve fibres predisposes to delayed corneal epithelial healing, reduced corneal sensitivity and corneal erosion. We investigated to what extent a reduction in corneal nerve fibre length (CNFL) is present in individuals with prediabetes or type 2 diabetes (DM2) compared with individuals with normal glucose metabolism (NGM).

**Methods:**

Using composite images acquired by corneal confocal microscopy, we assessed total CNFL per mm^2^ in the subbasal nerve plexus of the cornea in 134 participants (mean age 59 ± 8 years, 49% men, 87 NGM, 20 prediabetes, 27 DM2). Multivariable linear regression was used to assess the association between CNFL and glucose metabolism status, adjusted for age and sex.

**Results:**

In individuals with type 2 diabetes, the mean CNFL was significantly reduced [β = −1.86 mm/mm^2^ (95% CI −3.64 to −0.08), p = 0.04], as compared with individuals with normal glucose metabolism after adjustment for age and sex. Part of the reduction was present in individuals with prediabetes [β = −0.96 mm/mm^2^ (95% CI −2.91 to 0.99), p = 0.34], with a linear trend of corneal nerve fibre reduction with severity of glucose metabolism status (p trend = 0.04).

**Conclusions:**

A significant reduction in CNFL was found in individuals with DM2 compared with individuals with NGM. A trend of reduction in CNFL was observed between individuals with NGM and prediabetes. The reduction in corneal nerve fibre length could contribute to a delayed corneal healing and an increased risk for corneal complications after surgery.

## Introduction

The cornea is one of the body's most densely innervated tissues (Millodot 1984). Corneal nerves have important protective and trophic influences on the cornea (Muller et al. 2003). Under physiologic conditions, they play an important role in corneal epithelial cell metabolism, cell adhesion and wound healing in response to infection, trauma and surgery (Beuerman & Schimmelpfennig 1980; Linna et al. 1998; Muller et al. 2003; Gallar et al. 2004). Although one of the major ocular complications of diabetes is retinopathy, structural changes in the subbasal nerve plexus of the cornea are also observed (Rosenberg et al. 2000; Malik et al. 2003; Efron 2011). In individuals with diabetes, injury of the corneal nerve fibres results in a reduced tear secretion (Dogru et al. 2001; Saito et al. 2003; Yoon et al. 2004; Cousen et al. 2007), a delayed corneal epithelial healing (Kabosova et al. 2003) and a decreased corneal sensitivity (Nielsen & Lund 1979; Murphy et al. 2004; Tavakoli et al. 2007). Consequently, individuals with diabetes have a higher vulnerability for keratopathy (Schultz et al. 1983; Herse 1988; Ohashi 1997; Didenko et al. 1999), recurrent corneal erosions, persistent epithelial defects and neurotrophic corneal ulcers (Hyndiuk et al. 1977; Sanchez‐Thorin 1998). Since damage to the corneal nerves occurs in ocular and systemic diseases, and after corneal surgery (Patel & McGhee 2009), structural changes in the corneal plexus in individuals with glucose dysregulation can further jeopardize corneal epithelial healing (Beuerman & Schimmelpfennig 1980). In addition, corneal neurodegenerative changes in individuals with diabetes are correlated with diabetic polyneuropathy (Tavakoli et al. 2010; Dehghani et al. 2014; Ziegler et al. 2014; Ziegler et al. 2014; Pritchard et al. 2015). As signs of neuronal dysfunction are not only found in individuals with diabetes, but also found in individuals with prediabetes (Papanas et al. 2011; Bongaerts et al. 2013; Ziegler et al. 2014; Ziegler et al. 2014), neurodegenerative changes in the cornea could also be present before diabetes is clinically diagnosed (Asghar et al. 2014).

Corneal confocal microscopy (CCM) can accurately quantify the nerve fibre length in the subbasal nerve plexus of the cornea (Efron et al. 2010; Hertz et al. 2011; Petropoulos et al. 2013). Corneal confocal microscopy has a proven utility in detecting and monitoring neurodegenerative changes in individuals with diabetes (De Clerck et al. 2015). However, previous studies mainly included individuals with long‐duration type 2 diabetes (DM2). Since duration of diabetes is related to severity of diabetic neuropathy (Dyck et al. 1993), it would be of interest to assess changes in corneal nerve fibre length per mm^2^ (CNFL) in individuals with prediabetes. Establishing a reduction in CNFL in individuals with type 2 diabetes or prediabetes using CCM could help to identify individuals at risk for a delayed corneal healing (e.g. after routine cataract surgery), neurotrophic corneal ulcers and polyneuropathy at an earlier stage.

The aim of the present study was to investigate to what extent changes in CNFL are present in individuals with prediabetes and individuals with DM2, as compared to individuals with normal glucose metabolism (NGM).

## Materials and methods

### Study population and design

We used data from The Maastricht Study, an observational prospective population‐based cohort study. The rationale and methodology have been described previously (Schram et al. 2014). In brief, the study focuses on the aetiology, pathophysiology, complications and comorbidities of DM2 and is characterized by an extensive phenotyping approach. Eligible for participation were all individuals aged between 40 and 75 years and living in the southern part of the Netherlands. Participants were recruited through mass media campaigns and from the municipal registries and the regional Diabetes Patient Registry via mailings. Recruitment was stratified according to known DM2 status, with an oversampling of individuals with DM2, for reasons of efficiency. Cross‐sectional data of The Maastricht Study are available from the participants who completed the baseline survey between November 2010 and September 2013. From April 2013, corneal confocal microscopy measurements were included in the measurement protocol. The present report includes cross‐sectional data from all participants who completed the baseline survey between April 2013 and September 2013. The examinations of each participant were performed within a time window of 3 months. The study has been approved by the institutional medical ethical committee (NL31329.068.10) and the Minister of Health, Welfare and Sports of the Netherlands (Permit 131088‐105234‐PG). All participants gave written informed consent.

### Glucose metabolism status

To determine glucose metabolism, all participants, except those who used insulin, underwent a standardized 2‐h 75 g oral glucose tolerance test (OGTT) after an overnight fast. For safety reasons, participants with a fasting glucose level above 11.0 mmol/L, as determined by a finger prick, did not undergo the OGTT. For these individuals (*n* = 13), fasting glucose level and information about diabetes medication were used to determine glucose metabolism status. Glucose metabolism was defined according to the WHO criteria into NGM, impaired fasting glucose (IFG), impaired glucose tolerance (IGT), prediabetes (i.e. IFG and/or IGT), and DM2 (World Health Organization 2006). For this study, individuals with DM1, individuals with latent autoimmune diabetes of adults, steroid‐induced diabetes and individuals who underwent a pancreas transplantation were excluded.

### Ophthalmologic measurements

Corneal confocal microscopy (Heidelberg Retina Tomograph III, Rostock cornea module, Heidelberg Engineering, Heidelberg, Germany) was performed on the left eye to study the subbasal nerve plexus. Both eyes were anaesthetized with oxybuprocaine hydrochloride 0.4%. Both corneas were wetted with gel to prevent dry eyes and to ensure optimal contact between the cornea and the applanating cap. Participants were asked to fixate on a red light throughout the scan. The cornea was lightly touched with a TomoCap (Heidelberg Engineering) filled with gel. A charge‐coupled device camera was used to image the cornea from the side to check the correct position of the cap on the apex of the cornea. A joystick was used to identify the subbasal nerve plexus, located between the basal cell layer of the epithelium and the Bowman's layer. Images were acquired in the central part of the cornea by trained research assistants according to a standard operating procedure. The recorded images were composites of multiple smaller recordings of 400 × 400 μm (384 × 384 pixels, 8 bit) assembled together by the use of a composite algorithm implemented in the HRT3 user interface (Heidelberg Engineering, Heidelberg, Germany), as previously described (Allgeier et al. 2011; Allgeier et al. 2014). Real‐time mapping was performed on an area up to 1600 × 1600 µm (1536 × 1536 pixels, 8 bit), partially including the inferior whorl in some of the composite images. No measures were taken to include or exclude this region.

One two‐dimensional image was acquired in each participant. The examination lasted approximately 5 min. Corneal confocal microscopy measurements were not performed in individuals with a corneal infection. All images were reviewed individually, and their quality was scored based on the contrast, the depth and the sharpness of the picture and based on the presence of epithelial cells, Langerhans cells or pressure lines using a designated protocol. The area of the composite image was automatically assessed, the nerves in each image were automatically segmented, and the CNFL, defined as the total length of all nerve fibres with respect to the image area and expressed in mm per mm^2^, was measured by the use of a custom‐made software with a performance level comparable to a human grader (Guimaraes et al. 2016). Reproducibility was assessed by two observers in the left eye of six individuals (3 men, 64.5 ± 14.7 years; 1 individual with DM2) who were examined on two occasions with a 1‐week interval. The intra‐observer intra‐class correlation coefficients were ≥0.89, and the inter‐observer intra‐class correlation coefficients were ≥0.97.

The presence of corneal disorders, previous surgery and the use of contact lenses were assessed by questionnaire. In addition, the presence of an intraocular lens was assessed on the Scheimpflug image of the anterior segment (Oculus Pentacam HR, Wetzlar, Germany). Participants were requested to bring all medication they used or a list from their pharmacists to the research centre. Dry eye medication use and medication for corneal infection were assessed during a medication interview where generic name, dose and frequency were registered by trained staff (Schram et al. 2014). Individuals with a small investigated area (<0.50 mm^2^), poor quality imaging, corneal disorders, contact lenses, previous cataract or refractive surgery, and medication for dry eyes or corneal infection were excluded.

### Statistical analysis

Statistical analysis was performed in SPSS Statistics 23 for Windows; SPSS, Chicago, IL. Differences between group characteristics were tested using one‐way analysis of variance (anova) with post hoc testing by the least significant difference (LSD) method for continuous variables and chi‐square tests for categorical variables. Multivariable linear regression was used to analyse the association between glucose metabolism status (prediabetes and DM2; determinant) and CNFL (outcome). We combined the categories impaired fasting glucose (IFG) and impaired glucose tolerance (IGT) into prediabetes, because analyses did not show differences between IFG and IGT (data not shown). First, a crude analysis was performed. Next, associations were adjusted for age and sex. The results were expressed as regression coefficients (β), representing the mean difference in CNFL as compared with NGM, with their 95% confidence intervals (95% CIs) and p‐values. The Wilcoxon–Mann–Whitney 2‐tailed test was used for statistical power calculation to compare the CNFL in individuals with prediabetes versus individuals with NGM. Due to insufficient group size, the statistical power of 80% was not achieved.

## Results

**Figure 1:**
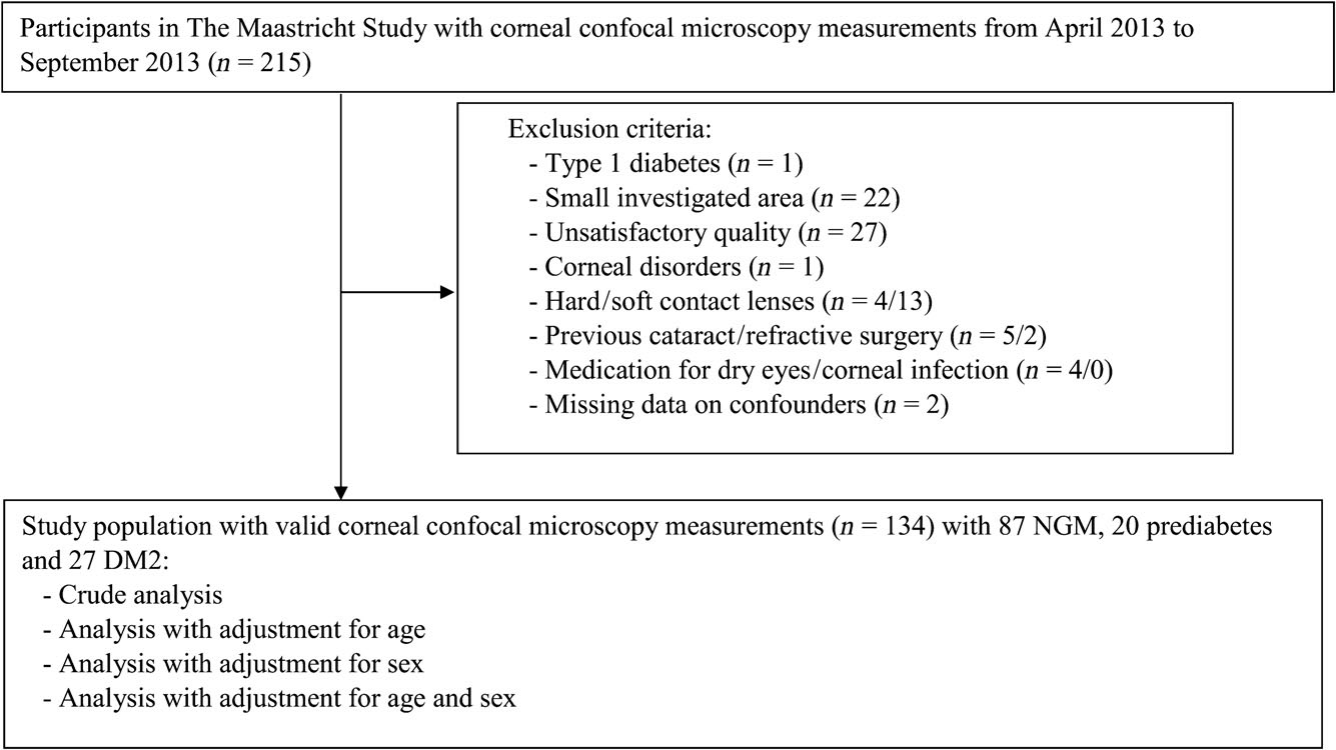
Flow diagram of the study. DM2 = type 2 diabetes, NGM = normal glucose metabolism.

Figure 1 shows the flow diagram of the study. A total of 215 consecutive participants had a corneal confocal microscopy measurement between April 2013 and September 2013. One participant with DM1 was excluded. Participants with a small investigated area of the subbasal nerve plexus were also excluded (*n* = 22). We additionally excluded images of unsatisfactory quality (*n* = 27) – that is unsatisfactory contrast (*n* = 5) or depth (*n* = 16), and/or the presence of epithelial cells (*n* = 9), disruptive Langerhans cells (*n* = 3) or pressure lines (*n* = 11). In addition, individuals with corneal disorders (*n* = 1), contact lenses (*n* = 17), previous cataract or refractive surgery (*n* = 7), and/or medication for dry eyes or corneal infection (*n* = 4) were also excluded. We additionally excluded individuals in whom data on ocular disorders, previous surgery or use of contact lenses were missing (*n* = 2). Thus, 134 participants were available for statistical analysis. Participants who were excluded due to missing values did not differ significantly from included participants in terms of age or sex.

**Figure 2:**
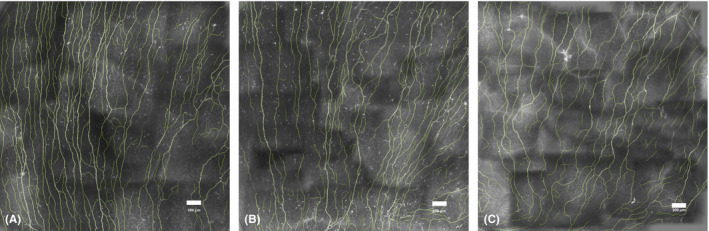
Corneal confocal microscopy recording of the subbasal nerve plexus using a composite beta software, showing nerves detected by our automated software used to calculate corneal nerve fibre length (mm/mm^2^) in an individual with a normal glucose metabolism (A), prediabetes (B) and type 2 diabetes (C).

General characteristics and the CNFL as assessed by CCM are shown in Table 1, stratified by glucose metabolism status. Of the 134 participants, 87 participants had NGM (64.9%), 20 participants had prediabetes (14.9%), and 27 participants had DM2 (20.1%). There was a statistically significant difference in age between individuals with DM2 and individuals with NGM (p < 0.01). In individuals with NGM, prediabetes and DM2, the crude CNFL was 13.4 ± 4.01 mm/mm^2^ [95% CI 12.5–14.3], 12.4 ± 3.02 mm/mm^2^ [95% CI 11.0–13.9], and 11.5 ± 4.11 mm/mm^2^ [95% CI 9.9–13.1], respectively (Fig. 2). The CNFL value was significantly lower in individuals with DM2 compared with individuals with NGM (p = 0.03), and a major part of this reduction was also present in prediabetes (p = 0.33). The mean investigated area of the composite images was 1.58 ± 0.66 mm^2^ in individuals with NGM, 1.78 ± 0.73 mm^2^ in individuals with prediabetes and 1.44 ± 0.56 mm^2^ in individuals with DM2, and was not significantly different between these groups (p = 0.22).

**Table 1:** Baseline characteristics of the study population and corneal nerve fibre length, stratified by glucose metabolism status.

	NGM (*n* = 87)	Prediabetes (*n* = 20)	DM2 (*n* = 27)	p‐value (Prediabetes vs NGM)	p‐value (DM2 vs NGM)
Age (years), mean ± SD	57.2 ± 8.1	61.0 ± 7.6	62.4 ± 8.5	0.07	<0.01*
Male sex, *n* (%)	40 (46.0)	9 (45.0)	17 (63.0)	0.94	0.12
Diabetes duration (years), median (IQR)†	–	–	0.0 (0.0–8.0)	–	–
Mean corneal nerve fibre length (mm/mm^2^), mean ± SD (95% CI)	13.4 ± 4.01 (12.5–14.3)	12.4 ± 3.02 (11.0–13.9)	11.5 ± 4.11 (9.9–13.1)*	0.33	0.03*

p‐values represent values obtained with one‐way analysis of variance with least significant difference (LSD) post hoc test for continuous variables and chi‐square tests for categorical variables.

DM2 = type 2 diabetes, NGM = normal glucose metabolism.

* p < 0.05 compared with NGM.

^†^ Available for 22 participants with DM2.

**Figure 3:**
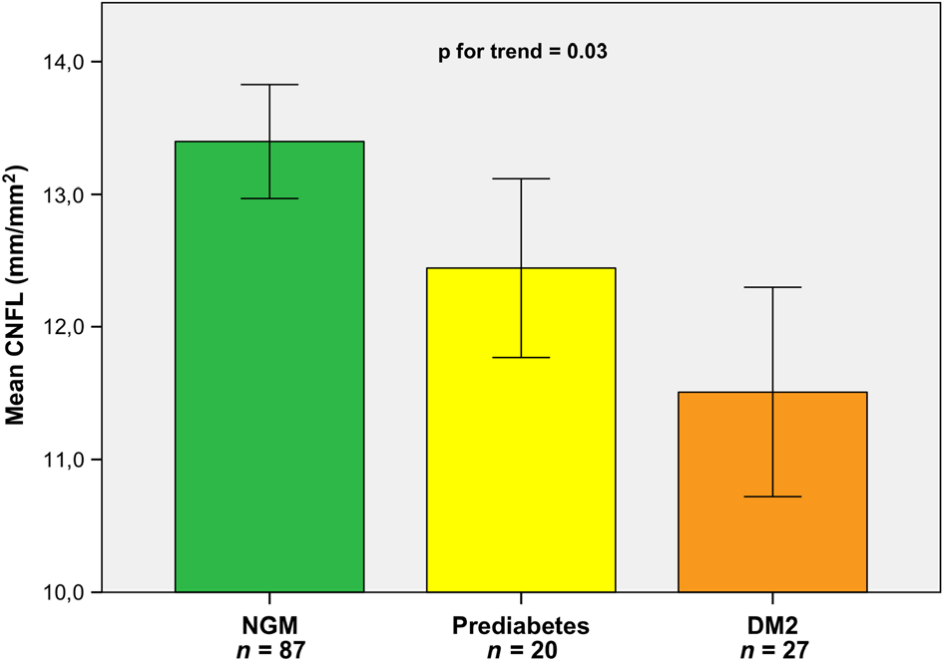
Crude corneal nerve fibre length for individuals with NGM, prediabetes and DM2 (Mean ± SE). CNFL = corneal nerve fibre length in mm per mm^2^, DM2 = type 2 diabetes, NGM = normal glucose metabolism.

Figure 3 shows crude CNFL values according to glucose metabolism status. Reduction in CNFL was more pronounced with worsening of glucose metabolism status, with a significant p‐value for linear trend (p = 0.03).

The CNFL decreased non‐significantly by −0.03 mm/mm^2^ [95% CI −0.11 to 0.06, p = 0.54] per one year of age and was also not significantly different between women and men [β = +0.33 mm/mm^2^ (95% CI −1.02 to +1.69), p = 0.63].

Table 2 shows crude, age‐ and sex‐adjusted associations between glucose metabolism status and CNFL. After adjustment for age and sex, individuals with prediabetes [β = −0.96 mm/mm^2^ (95% CI −2.91 to 0.99), p = 0.34] and individuals with DM2 [β = −1.86 mm/mm^2^ (95% CI −3.64 to −0.08), p = 0.04] showed lower CNFL values as compared with individuals with NGM. The reduction in CNFL was more pronounced with worsening of glucose metabolism status, with a statistically significant p‐value for linear trend (p = 0.04).

**Table 2:** Mean difference of corneal nerve fibre length between individuals with prediabetes and type 2 diabetes versus normal glucose metabolism, adjusted for age and sex.

	Corneal nerve fibre length (mm/mm^2^)				
	Prediabetes (*n* = 20)		Type 2 diabetes (*n* = 27)		Trend
	β (95% CI)	p	β (95% CI)	p	p
Crude analysis	−0.96 (−2.87 to 0.96)	0.33	−1.89 (−3.59 to −0.19)	0.03*	0.03*
Adjustment for age	−0.95 (−2.90 to 0.99)	0.34	−1.88 (−3.64 to −0.12)	0.04*	0.03*
Adjustment for sex	−0.96 (−2.88 to 0.96)	0.33	−1.86 (−3.58 to −0.14)	0.03*	0.03*
Adjustment for age and sex	−0.96 (−2.91 to 0.99)	0.34	−1.86 (−3.64 to −0.08)	0.04*	0.04*

Multivariable linear regression was used to analyse the association between glucose metabolism status and CNFL.

β = regression coefficient, 95% CI = 95% confidence interval, p = p‐value.

* p < 0.05.

## Discussion

In the current study, we showed a significant reduction in CNFL between individuals with DM2 compared with individuals with NGM using composite CCM images. Our findings suggest a trend of reduction in CNFL from NGM to prediabetes.

To our knowledge, this is the first study that uses composite CCM images to assess the association between glucose metabolism status, as defined by an oral glucose tolerance test, and CNFL. This innovative imaging technique based on real‐time mapping of several images allows visualization of a larger continuous surface of the corneal subbasal nerve plexus, increasing the level of accuracy of quantification of CNFL (Patel & McGhee 2005; Zhivov et al. 2010; Vagenas et al. 2012; Lagali et al. 2018). The method used for the assessment of the CNFL in these images showed a high reproducibility. Standard deviations were in line with those reported in previous studies. Larger study populations are needed to confirm our findings.

In individuals with NGM, the mean CNFL was 13.4 mm/mm^2^, which is in line with previous studies reporting a mean CNFL in the range of 11.90–24.30 mm/mm^2^ (Hertz et al. 2011; Ahmed et al. 2012; Nitoda et al. 2012; Sivaskandarajah et al. 2013; Bitirgen et al. 2014; Dehghani et al. 2014; Pritchard et al. 2014; Stem et al. 2014; Ziegler et al. 2014; Ziegler et al. 2014; Lagali et al. 2017). In individuals with prediabetes, the mean CNFL was 12.4 mm/mm^2^. At present, only three studies assessed corneal neurodegenerative variables in individuals with prediabetes, reporting a mean CNFL of 14.6, 15.3 and 22.1 mm/mm^2^ (Asghar et al. 2014; Azmi et al. 2015; Lagali et al. 2017, respectively). In individuals with DM2 in our study, the mean CNFL was 11.5 mm/mm^2^, which is in line with previous studies reporting a mean CNFL in the range of 9.80–21.90 mm/mm^2^ (Nitoda et al. 2012; Bitirgen et al. 2014; Stem et al. 2014; Ziegler et al. 2014; Ziegler et al. 2014; Lagali et al. 2017). The large range in CNFL in previous studies could be explained by heterogeneity in terms of type of in vivo confocal microscope, scan protocol, image selection and software for analysis of the CNFL. In our study, the CNFL in DM2 was 14% reduced compared with individuals with NGM. This is in line with the findings of previous studies, showing a reduction by 8–18% without correction for age and sex (Nitoda et al. 2012; Bitirgen et al. 2014; Stem et al. 2014; Ziegler et al. 2014; Ziegler et al. 2014; Lagali et al. 2017). However, these previous studies mainly included individuals with long‐duration DM2. Our study showed no significant influence of age on the CNFL (−0.03 mm/mm^2^ per year, p = 0.54), which is similar to the findings of the LANDMARK study (−0.05 mm/mm^2^ per year, p > 0.05) (Edwards et al. 2012). Another study found a significant negative correlation between age and CNFL, showing a mean decline of 0.25–0.30% per year, which agrees with the reduction of 0.22% per year found in our study (Parissi et al. 2013). Similar to previous studies, the CNFL was not significantly different between men and women (Edwards et al. 2012; Parissi et al. 2013).

Diabetes induces alterations of the corneal subbasal nerve plexus, such as a reduction in the CNFL assessed by CCM. However, the exact pathogenic mechanisms underlying the diabetes‐associated reduction in CNFL are not clear. Corneal neuropathy has been suggested to be caused by paracrine signals reaching the cornea via the limbal capillaries (Leppin et al. 2014). Accumulation of advanced glycation end products (Reichard 2012), impaired endothelium‐dependent vasodilation (Davidson et al. 2012), altered release of neuropeptides (Leppin et al. 2014) and downregulation of neurotrophic factors (Muller et al. 2003), could play a role in the pathogenesis of corneal neuropathy. This downregulation of neurotrophic factors may result in an impaired epithelial healing capacity in individuals with diabetes (You et al. 2000; Cursiefen et al. 2005).

Delayed corneal epithelial wound healing after subbasal nerve plexus injuries following refractive surgery, corneal transplantation, cataract surgery and vitrectomy leads to an increased incidence of postoperative epithelial complications and poorer refractive results (Foulks et al. 1979; Fraunfelder & Rich 2002). Postoperative regeneration of the subbasal nerves is a long‐lasting process, which generally does not recover to normal, even in individuals with NGM. After laser‐assisted in situ keratomileusis (LASIK) (Erie et al. 2005), laser epithelial keratomileusis (LASEK) (Darwish et al. 2007) and photorefractive keratectomy (PRK) (Moilanen et al. 2003; Erie et al. 2005) healing periods up to five years have been reported, and after penetrating keratoplasty (PKP) (Niederer et al. 2007), structural changes in the subbasal nerves remain visible even 40 years after surgery. In addition, structural changes in the subbasal nerve plexus have been observed 6 months after phacoemulsification with intraocular lens insertion (Misra et al. 2015). Finally, a delayed re‐innervation after corneal epithelial erosions has been observed in DM2 (Wang et al. 2012).

A trend of reduction in CNFL is now observed from NGM to prediabetes. Because a reduction in CNFL is associated with a delayed corneal epithelial healing (Kabosova et al. 2003) and a predisposition for corneal ulcers and other corneal complications, in particular after surgery (Hyndiuk et al. 1977; Sanchez‐Thorin 1998; Patel & McGhee 2009), we could suppose that this group of individuals may also be at risk. Since diabetes is a contra‐indication for corneal laser refractive surgery (Netherlands Ophthalmologic Society 2013), one could hypothesize that individuals with prediabetes also present a relative systemic contra‐indication for ocular surgery. Early diagnosis of prediabetes by the oral glucose tolerance test and the assessment of corneal neurodegenerative changes by corneal confocal microscopy may define patients at risk for corneal neuropathy. In patients with corneal neuropathy, the risk for postoperative epithelial complications could be reduced by increased lubrication and management of meibomian gland dysfunction (Krachmer 2011; Weisenthal et al. 2017). In addition, surgical epithelial debridement should be minimized and toxic medications should be avoided. Furthermore, the early observation of CNFL reduction, which is an ophthalmic marker of polyneuropathy (Ahmed et al. 2012; Sivaskandarajah et al. 2013; Dehghani et al. 2014; Pritchard et al. 2015), is in line with previous studies demonstrating neuropathic symptoms (Isak et al. 2008; Ziegler et al. 2009; Asghar et al. 2014), abnormal (semi‐) quantitative sensory tests (Franklin et al. 1990; Asghar et al. 2014) and reduction in intraepidermal nerve fibre density in skin biopsy samples in individuals with prediabetes (Smith et al. 2006; Asghar et al. 2014; Azmi et al. 2015).

There are several limitations of the present study. First, the CNFL is currently the only variable calculated by our software. However, the CNFL is known to be the corneal neuronal variable with the best reproducibility compared with other corneal neuronal variables (Efron et al. 2010; Hertz et al. 2011; Petropoulos et al. 2013). Second, the cross‐sectional design does not allow to address causal relationships. However, by comparing individuals with NGM, prediabetes and DM2, we mimic the pathological pathway of glucose metabolism deterioration. Third, we did not perform a slit‐lamp examination and did not assess ocular sensitivity in our study. Future longitudinal studies should focus on causality and underlying mechanisms of the decrease in CNFL in prediabetes and DM2 and its predictive value for corneal pathology.

In conclusion, this study demonstrates that a significant reduction in CNFL is found in individuals with DM2 compared with individuals with NGM. A trend of reduction is observed between individuals with NGM and prediabetes. This may result in a delayed corneal healing and an increased risk for corneal complications after surgery.

## References

[aos14359-bib-0001] Ahmed A , Bril V , Orszag A , Paulson J , Yeung E , Ngo M , Orlov S & Perkins BA (2012): Detection of diabetic sensorimotor polyneuropathy by corneal confocal microscopy in type 1 diabetes: a concurrent validity study. Diabetes Care 35: 821–828. 22323412 10.2337/dc11-1396 PMC3308301

[aos14359-bib-0002] Allgeier S , Zhivov A , Eberle F , Koehler B , Maier S , Bretthauer G , Guthoff RF & Stachs O (2011): Image reconstruction of the subbasal nerve plexus with in vivo confocal microscopy. Invest Ophthalmol Vis Sci 52: 5022–5028. 21447691 10.1167/iovs.10-6065

[aos14359-bib-0003] Allgeier S , Maier S , Mikut R , Peschel S , Reichert KM , Stachs O & Kohler B (2014): Mosaicking the subbasal nerve plexus by guided eye movements. Invest Ophthalmol Vis Sci 55: 6082–6089. 25159207 10.1167/iovs.14-14698

[aos14359-bib-0004] Asghar O , Petropoulos IN , Alam U et al. (2014): Corneal confocal microscopy detects neuropathy in subjects with impaired glucose tolerance. Diabetes Care 37: 2643–2646. 24969581 10.2337/dc14-0279 PMC4140158

[aos14359-bib-0005] Azmi S , Ferdousi M , Petropoulos IN et al. (2015): Corneal confocal microscopy identifies small‐fiber neuropathy in subjects with impaired glucose tolerance who develop type 2 diabetes. Diabetes Care 38: 1502–1508. 25877814 10.2337/dc14-2733 PMC4512140

[aos14359-bib-0006] Beuerman RW & Schimmelpfennig B (1980): Sensory denervation of the rabbit cornea affects epithelial properties. Exp Neurol 69: 196–201. 7389846 10.1016/0014-4886(80)90154-5

[aos14359-bib-0007] Bitirgen G , Ozkagnici A , Malik RA & Kerimoglu H (2014): Corneal nerve fibre damage precedes diabetic retinopathy in patients with type 2 diabetes mellitus. Diabetes Med 31: 431–438. 10.1111/dme.12324 24117485

[aos14359-bib-0008] Bongaerts BW , Rathmann W , Heier M , Kowall B , Herder C , Stockl D , Meisinger C & Ziegler D (2013): Older subjects with diabetes and prediabetes are frequently unaware of having distal sensorimotor polyneuropathy: the KORA F4 study. Diabetes Care 36: 1141–1146. 23275355 10.2337/dc12-0744 PMC3631873

[aos14359-bib-0009] Cousen P , Cackett P , Bennett H , Swa K & Dhillon B (2007): Tear production and corneal sensitivity in diabetes. J Diabetes Complications 21: 371–373. 17967709 10.1016/j.jdiacomp.2006.05.008

[aos14359-bib-0010] Cursiefen C , Seitz B & Kruse FE (2005): Neurotrophic keratitis. Der Ophthalmologe 102: 7–14. 15538584 10.1007/s00347-004-1140-z

[aos14359-bib-0011] Darwish T , Brahma A , O'Donnell C & Efron N (2007): Subbasal nerve fiber regeneration after LASIK and LASEK assessed by noncontact esthesiometry and in vivo confocal microscopy: prospective study. J Cataract Refract Surg 33: 1515–1521. 17720064 10.1016/j.jcrs.2007.05.023

[aos14359-bib-0012] Davidson EP , Coppey LJ , Holmes A & Yorek MA (2012): Changes in corneal innervation and sensitivity and acetylcholine‐mediated vascular relaxation of the posterior ciliary artery in a type 2 diabetic rat. Invest Ophthalmol Vis Sci 53: 1182–1187. 22273725 10.1167/iovs.11-8806 PMC3339902

[aos14359-bib-0013] De Clerck EE , Schouten JS , Berendschot TT et al. (2015): New ophthalmologic imaging techniques for detection and monitoring of neurodegenerative changes in diabetes: a systematic review. Lancet Diabetes Endocrinol 3: 653–663. 26184671 10.1016/S2213-8587(15)00136-9

[aos14359-bib-0014] Dehghani C , Pritchard N , Edwards K , Vagenas D , Russell AW , Malik RA & Efron N (2014): Natural history of corneal nerve morphology in mild neuropathy associated with type 1 diabetes: development of a potential measure of diabetic peripheral neuropathy. Invest Ophthalmol Vis Sci 55: 7982–7990. 25406279 10.1167/iovs.14-15605

[aos14359-bib-0015] Didenko TN , Smoliakova GP , Sorokin EL & Egorov VV (1999): Clinical and pathogenetic features of neurotrophic corneal disorders in diabetes. Vestn oftalmol 115: 7–11. 10665277

[aos14359-bib-0016] Dogru M , Katakami C & Inoue M (2001): Tear function and ocular surface changes in noninsulin‐dependent diabetes mellitus. Ophthalmology 108: 586–592. 11237914 10.1016/s0161-6420(00)00599-6

[aos14359-bib-0017] Dyck PJ , Kratz KM , Karnes JL et al. (1993): The prevalence by staged severity of various types of diabetic neuropathy, retinopathy, and nephropathy in a population‐based cohort: the Rochester Diabetic Neuropathy Study. Neurology 43: 817–824. 8469345 10.1212/wnl.43.4.817

[aos14359-bib-0018] Edwards K , Pritchard N , Vagenas D , Russell A , Malik RA & Efron N (2012): Utility of corneal confocal microscopy for assessing mild diabetic neuropathy: baseline findings of the LANDMark study. Clin Exp Optom 95: 348–354. 22540156 10.1111/j.1444-0938.2012.00740.x

[aos14359-bib-0019] Efron N (2011): The Glenn A. Fry award lecture 2010: Ophthalmic markers of diabetic neuropathy. Optom Vis Sci 88: 661–683. 21478787 10.1097/OPX.0b013e3182171020

[aos14359-bib-0020] Efron N , Edwards K , Roper N et al. (2010): Repeatability of measuring corneal subbasal nerve fiber length in individuals with type 2 diabetes. Eye Contact Lens 36: 245–248. 20724854 10.1097/ICL.0b013e3181eea915

[aos14359-bib-0021] Erie JC , McLaren JW , Hodge DO & Bourne WM (2005): Recovery of corneal subbasal nerve density after PRK and LASIK. Am J Ophthalmol 140: 1059–1064. 16376651 10.1016/j.ajo.2005.07.027

[aos14359-bib-0022] Foulks GN , Thoft RA , Perry HD & Tolentino FI (1979): Factors related to corneal epithelial complications after closed vitrectomy in diabetics. Arch Ophthalmol 97: 1076–1078. 444136 10.1001/archopht.1979.01020010530002

[aos14359-bib-0023] Franklin GM , Kahn LB , Baxter J , Marshall JA & Hamman RF (1990): Sensory neuropathy in non‐insulin‐dependent diabetes mellitus. The San Luis Valley Diabetes Study. Am J Epidemiol 131: 633–643. 2316495 10.1093/oxfordjournals.aje.a115547

[aos14359-bib-0024] Fraunfelder FW & Rich LF (2002): Laser‐assisted in situ keratomileusis complications in diabetes mellitus. Cornea 21: 246–248. 11917170 10.1097/00003226-200204000-00002

[aos14359-bib-0025] Gallar J , Acosta MC , Moilanen JA , Holopainen JM , Belmonte C & Tervo TM (2004): Recovery of corneal sensitivity to mechanical and chemical stimulation after laser in situ keratomileusis. J Refract Surg 20: 229–235. 15188899 10.3928/1081-597X-20040501-06

[aos14359-bib-0026] Guimaraes P , Wigdahl J & Ruggeri A (2016): A fast and efficient technique for the automatic tracing of corneal nerves in confocal microscopy. Transl Vis Sci Technol 5: 7. 10.1167/tvst.5.5.7 PMC5054765 27730007

[aos14359-bib-0027] Herse PR (1988): A review of manifestations of diabetes mellitus in the anterior eye and cornea. Am J Optom Physiol Opt 65: 224–230. 3284372 10.1097/00006324-198803000-00013

[aos14359-bib-0028] Hertz P , Bril V , Orszag A , Ahmed A , Ng E , Nwe P , Ngo M & Perkins BA (2011): Reproducibility of in vivo corneal confocal microscopy as a novel screening test for early diabetic sensorimotor polyneuropathy. Diabet Med 28: 1253–1260. 21434993 10.1111/j.1464-5491.2011.03299.x

[aos14359-bib-0029] Hyndiuk RA , Kazarian EL , Schultz RO & Seideman S (1977): Neurotrophic corneal ulcers in diabetes mellitus. Arch Ophthalmol 95: 2193–2196. 588113 10.1001/archopht.1977.04450120099012

[aos14359-bib-0030] Isak B , Oflazoglu B , Tanridag T , Yitmen I & Us O (2008): Evaluation of peripheral and autonomic neuropathy among patients with newly diagnosed impaired glucose tolerance. Diabetes Metab Res Rev 24: 563–569. 18636432 10.1002/dmrr.859

[aos14359-bib-0031] Kabosova A , Kramerov AA , Aoki AM , Murphy G , Zieske JD & Ljubimov AV (2003): Human diabetic corneas preserve wound healing, basement membrane, integrin and MMP‐10 differences from normal corneas in organ culture. Exp Eye Res 77: 211–217. 12873452 10.1016/s0014-4835(03)00111-8 PMC2909880

[aos14359-bib-0032] Krachmer JHMM & Holland EJ (2011): Cornea. Philadelphia: Elsevier/Mosby.

[aos14359-bib-0033] Lagali NS , Allgeier S , Guimaraes P et al. (2017): Reduced corneal nerve fiber density in type 2 diabetes by wide‐area mosaic analysis. Invest Ophthalmol Vis Sci 58: 6318–6327. 29242906 10.1167/iovs.17-22257

[aos14359-bib-0034] Lagali NS , Allgeier S , Guimaraes P et al. (2018): Wide‐field corneal subbasal nerve plexus mosaics in age‐controlled healthy and type 2 diabetes populations. Sci Data 5: 180075. 29688226 10.1038/sdata.2018.75 PMC5914299

[aos14359-bib-0035] Leppin K , Behrendt AK , Reichard M , Stachs O , Guthoff RF , Baltrusch S , Eule JC & Vollmar B (2014): Diabetes mellitus leads to accumulation of dendritic cells and nerve fiber damage of the subbasal nerve plexus in the cornea. Invest Ophthalmol Vis Sci 55: 3603–3615. 24781935 10.1167/iovs.14-14307

[aos14359-bib-0036] Linna TU , Perez‐Santonja JJ , Tervo KM , Sakla HF , Alió y Sanz JL & Tervo TM (1998): Recovery of corneal nerve morphology following laser in situ keratomileusis. Exp Eye Res 66: 755–763. 9657908 10.1006/exer.1998.0469

[aos14359-bib-0037] Malik RA , Kallinikos P , Abbott CA , van Schie CH , Morgan P , Efron N & Boulton AJ (2003): Corneal confocal microscopy: a non‐invasive surrogate of nerve fibre damage and repair in diabetic patients. Diabetologia 46: 683–688. 12739016 10.1007/s00125-003-1086-8

[aos14359-bib-0038] Millodot M (1984): A review of research on the sensitivity of the cornea. Ophthalmic Physiol Opt 4: 305–318. 6390296

[aos14359-bib-0039] Misra SL , Goh YW , Patel DV , Riley AF & McGhee CN (2015): Corneal microstructural changes in nerve fiber, endothelial and epithelial density after cataract surgery in patients with diabetes mellitus. Cornea 34: 177–181. 25474233 10.1097/ICO.0000000000000320

[aos14359-bib-0040] Moilanen JA , Vesaluoma MH , Muller LJ & Tervo TM (2003): Long‐term corneal morphology after PRK by in vivo confocal microscopy. Invest Ophthalmol Vis Sci 44: 1064–1069. 12601030 10.1167/iovs.02-0247

[aos14359-bib-0041] Muller LJ , Marfurt CF , Kruse F & Tervo TM (2003): Corneal nerves: structure, contents and function. Exp Eye Re 76: 521–542. 10.1016/s0014-4835(03)00050-2 12697417

[aos14359-bib-0042] Murphy PJ , Patel S , Kong N , Ryder RE & Marshall J (2004): Noninvasive assessment of corneal sensitivity in young and elderly diabetic and nondiabetic subjects. Invest Ophthalmol Vis Sci 45: 1737–1742. 15161834 10.1167/iovs.03-0689

[aos14359-bib-0043] Netherlands Ophthalmologic Society (2013): Consensus refractive surgery. http://www.ooglaseradvies.org/wp-content/uploads/2013/12/ConsensusRC2013.pdf.

[aos14359-bib-0044] Niederer RL , Perumal D , Sherwin T & McGhee CN (2007): Corneal innervation and cellular changes after corneal transplantation: an in vivo confocal microscopy study. Invest Ophthalmol Vis Sci 48: 621–626. 17251458 10.1167/iovs.06-0538

[aos14359-bib-0045] Nielsen NV & Lund FS (1979): Diabetic polyneuropathy. Corneal sensitivity, vibratory perception and Achilles tendon reflex in diabetics. Acta Neurol Scand 59: 15–22. 433574 10.1111/j.1600-0404.1979.tb02906.x

[aos14359-bib-0046] Nitoda E , Kallinikos P , Pallikaris A , Moschandrea J , Amoiridis G , Ganotakis ES & Tsilimbaris M (2012): Correlation of diabetic retinopathy and corneal neuropathy using confocal microscopy. Curr Eye Res 37: 898–906. 22632054 10.3109/02713683.2012.683507

[aos14359-bib-0047] Ohashi Y (1997): Diabetic keratopathy. Nippon Ganka Gakkai Zasshi 101: 105–110. 9124089

[aos14359-bib-0048] Papanas N , Vinik AI & Ziegler D (2011): Neuropathy in prediabetes: does the clock start ticking early? Nature reviews. Endocrinology 7: 682–690. 21750507 10.1038/nrendo.2011.113

[aos14359-bib-0049] Parissi M , Karanis G , Randjelovic S , Germundsson J , Poletti E , Ruggeri A , Utheim TP & Lagali N (2013): Standardized baseline human corneal subbasal nerve density for clinical investigations with laser‐scanning in vivo confocal microscopy. Invest Ophthalmol Vis Sci 54: 7091–7102. 24084094 10.1167/iovs.13-12999

[aos14359-bib-0050] Patel DV & McGhee CN (2005): Mapping of the normal human corneal sub‐Basal nerve plexus by in vivo laser scanning confocal microscopy. Invest Ophthalmol Vis Sci 46: 4485–4488. 16303938 10.1167/iovs.05-0794

[aos14359-bib-0051] Patel DV & McGhee CN (2009): In vivo confocal microscopy of human corneal nerves in health, in ocular and systemic disease, and following corneal surgery: a review. Br J Ophthalmol 93: 853–860. 19019923 10.1136/bjo.2008.150615

[aos14359-bib-0052] Petropoulos IN , Manzoor T , Morgan P et al. (2013): Repeatability of in vivo corneal confocal microscopy to quantify corneal nerve morphology. Cornea 32: e83–89. 23172119 10.1097/ICO.0b013e3182749419

[aos14359-bib-0053] Pritchard N , Edwards K , Dehghani C et al. (2014): Longitudinal assessment of neuropathy in type 1 diabetes using novel ophthalmic markers (LANDMark): study design and baseline characteristics. Diabetes Res Clin Pract 104: 248–256. 24629408 10.1016/j.diabres.2014.02.011

[aos14359-bib-0054] Pritchard N , Edwards K , Russell AW , Perkins BA , Malik RA & Efron N (2015): Corneal confocal microscopy predicts 4‐year incident peripheral neuropathy in type 1 diabetes. Diabetes Care 38: 671–675. 25573881 10.2337/dc14-2114

[aos14359-bib-0055] Reichard MWH , Waterstadt R , Tiedge M , Stachs O & Baltrusch S (2012): Manifestation of neuropathy in the corneal nerve plexus correlates with hyperglycemia and increased advanced glycation end products EASD.

[aos14359-bib-0056] Rosenberg ME , Tervo TM , Immonen IJ , Muller LJ , Gronhagen‐Riska C & Vesaluoma MH (2000): Corneal structure and sensitivity in type 1 diabetes mellitus. Invest Ophthalmol Vis Sci 41: 2915–2921. 10967045

[aos14359-bib-0057] Saito J , Enoki M , Hara M , Morishige N , Chikama T & Nishida T (2003): Correlation of corneal sensation, but not of basal or reflex tear secretion, with the stage of diabetic retinopathy. Cornea 22: 15–18. 12502941 10.1097/00003226-200301000-00004

[aos14359-bib-0058] Sanchez‐Thorin JC (1998): The cornea in diabetes mellitus. Int Ophthalmol Clin 38: 19–36. 9604736

[aos14359-bib-0059] Schram MT , Sep SJ , van der Kallen CJ , Dagnelie PC , Koster A , Schaper N , Henry RM & Stehouwer CD (2014): The Maastricht Study: an extensive phenotyping study on determinants of type 2 diabetes, its complications and its comorbidities. Eur J Epidemiol 29: 439–451. 24756374 10.1007/s10654-014-9889-0

[aos14359-bib-0060] Schultz RO , Peters MA , Sobocinski K , Nassif K & Schultz KJ (1983): Diabetic corneal neuropathy. Trans Am Ophthalmol Soc 81: 107–124. 6676964 PMC1312443

[aos14359-bib-0061] Sivaskandarajah GA , Halpern EM , Lovblom LE , Weisman A , Orlov S , Bril V & Perkins BA (2013): Structure‐function relationship between corneal nerves and conventional small‐fiber tests in type 1 diabetes. Diabetes Care 36: 2748–2755. 23579181 10.2337/dc12-2075 PMC3747893

[aos14359-bib-0062] Smith AG , Russell J , Feldman EL et al. (2006): Lifestyle intervention for pre‐diabetic neuropathy. Diabetes Care 29: 1294–1299. 16732011 10.2337/dc06-0224

[aos14359-bib-0063] Stem MS , Hussain M , Lentz SI , Raval N , Gardner TW , Pop‐Busui R & Shtein RM (2014): Differential reduction in corneal nerve fiber length in patients with type 1 or type 2 diabetes mellitus. J Diabetes Complications 28: 658–661. 25044236 10.1016/j.jdiacomp.2014.06.007 PMC4146399

[aos14359-bib-0064] Tavakoli M , Kallinikos PA , Efron N , Boulton AJ & Malik RA (2007): Corneal sensitivity is reduced and relates to the severity of neuropathy in patients with diabetes. Diabetes Care 30: 1895–1897. 17372147 10.2337/dc07-0175

[aos14359-bib-0065] Tavakoli M , Quattrini C , Abbott C et al. (2010): Corneal confocal microscopy: a novel noninvasive test to diagnose and stratify the severity of human diabetic neuropathy. Diabetes Care 33: 1792–1797. 20435796 10.2337/dc10-0253 PMC2909064

[aos14359-bib-0066] Vagenas D , Pritchard N , Edwards K , Shahidi AM , Sampson GP , Russell AW , Malik RA & Efron N (2012): Optimal image sample size for corneal nerve morphometry. Optometry Vis Sci 89: 812–817. 10.1097/OPX.0b013e31824ee8c9 22407254

[aos14359-bib-0067] Wang F , Gao N , Yin J & Yu FS (2012): Reduced innervation and delayed re‐innervation after epithelial wounding in type 2 diabetic Goto‐Kakizaki rats. Am J Pathol 181: 2058–2066. 23063510 10.1016/j.ajpath.2012.08.029 PMC3509759

[aos14359-bib-0068] Weisenthal RW, Bouchard CS , Colby KA , Rootman DS , Tu EY & de Freitas D (2017): Basic and Clinical Science Course (BCSC), 2016–2017: Section 8: External Disease and Cornea. San Francisco: American Academy of Ophthalmology.

[aos14359-bib-0069] World Health Organization (2006): Definition and diagnosis of diabetes mellitus and intermediate hyperglycemia: report of a WHO/IDF consultation. Geneva, Switzerland Published January 14, 2006. http://www.who.int/diabetes/publications/Definitionanddiagnosisofdiabetes_new.pdf

[aos14359-bib-0070] Yoon KC , Im SK & Seo MS (2004): Changes of tear film and ocular surface in diabetes mellitus. Korean J Ophthalmol 18: 168–174. 15635831 10.3341/kjo.2004.18.2.168

[aos14359-bib-0071] You L , Kruse FE & Volcker HE (2000): Neurotrophic factors in the human cornea. Invest Ophthalmol Vis Sci 41: 692–702. 10711683

[aos14359-bib-0072] Zhivov A , Blum M , Guthoff R & Stachs O (2010): Real‐time mapping of the subepithelial nerve plexus by in vivo confocal laser scanning microscopy. Br J Ophthalmol 94: 1133–1135. 20813752 10.1136/bjo.2009.175489

[aos14359-bib-0073] Ziegler D , Rathmann W , Dickhaus T , Meisinger C & Mielck A ; KS Group (2009): Neuropathic pain in diabetes, prediabetes and normal glucose tolerance: the MONICA/KORA Augsburg Surveys S2 and S3. Pain medicine 10: 393–400. 19207236 10.1111/j.1526-4637.2008.00555.x

[aos14359-bib-0074] Ziegler D , Papanas N , Vinik AI & Shaw JE (2014): Epidemiology of polyneuropathy in diabetes and prediabetes. Handbook Clin Neurol 126: 3–22. 10.1016/B978-0-444-53480-4.00001-1 25410210

[aos14359-bib-0075] Ziegler D , Papanas N , Zhivov A et al.; G German Diabetes Study (2014): Early detection of nerve fiber loss by corneal confocal microscopy and skin biopsy in recently diagnosed type 2 diabetes. Diabetes 63: 2454–2463. 24574045 10.2337/db13-1819

